# A dataset of formulation compositions for self-emulsifying drug delivery systems

**DOI:** 10.1038/s41597-023-02812-w

**Published:** 2023-12-20

**Authors:** Jonathan Zaslavsky, Christine Allen

**Affiliations:** 1 Leslie Dan Faculty of Pharmacy, University of Toronto, Toronto, ON M5S 3M2 Canada; 2 Department of Chemical Engineering & Applied Chemistry, University of Toronto, Toronto, ON M5S 3E5 Canada; 3 Acceleration Consortium, Toronto, ON M5S 3H6 Canada

**Keywords:** Drug delivery, Pharmaceutics, Preclinical research

## Abstract

Self-emulsifying drug delivery systems (SEDDS) are a well-established formulation strategy for improving the oral bioavailability of poorly water-soluble drugs. Traditional development of these formulations relies heavily on empirical observation to assess drug and excipient compatibility, as well as to select and optimize the formulation compositions. The aim of this work was to leverage previously developed SEDDS in the literature to construct a comprehensive SEDDS dataset that can be used to gain insights and advance data-driven approaches to formulation development. A dataset comprised of 668 unique SEDDS formulations encompassing 20 poorly water-soluble drugs was curated. While there are still opportunities to enhance the quality and quantity of data on SEDDS, this research lays the groundwork to potentially simplify the SEDDS formulation development process.

## Background & Summary

Poor aqueous solubility and permeability are recognized as major contributors to limited oral drug bioavailability. Indeed, these are integral considerations of theoretical frameworks such as Lipinski’s Rule of Five, the Biopharmaceutical Classification System (BCS), and the expanded Developability Classification System (DCS), which provide ways to differentiate promising drugs for oral administration^1–3^. Over time, it has been reported that a growing number of small molecule drug candidates exhibit properties that may hinder oral absorption. In fact, in the 20 years since the rule of five was first proposed, new chemical entities approved by the FDA have been shown to increase in molecular weight and calculated water-octanol partition coefficient (clogP)^4,5^. In general, the successful clinical approval of less traditionally drug-like molecules underscores the critical role of pharmaceutical formulations.

Advanced lipid-based formulation strategies have enabled enhancement of oral absorption of drugs with poor water solubility and/or low intestinal permeability (i.e., BCS II and IV drugs). One such example is self-emulsifying drug delivery systems (SEDDS), a combination of oils, surfactants, and/or cosolvents that spontaneously emulsify in the aqueous environment of the gastrointestinal tract^6^. The ability of SEDDS formulations to improve oral bioavailability has been attributed to a number of mechanisms, notably through increased apparent solubility of highly lipophilic drugs, as well as reduced metabolism or efflux^7^. As a result, several clinically approved drugs rely on delivery in SEDDS formulations including cyclosporine A (e.g., Sandimmune, Neoral), tipranavir (e.g., Aptivus), and fenofibrate (e.g., Lipofen), among others^8–10^.

Despite the relative simplicity of SEDDS in principle, the path to design such formulations remains non-trivial. The traditional approach to SEDDS development is an empirical process relying on iterative trial-and-error to screen, optimize, and evaluate the formulations. One of the most pertinent questions lies with the selection of appropriate excipients and mixtures thereof. Typically, this begins with quantification of the drug solubility in excipients, followed by screening excipient mixtures based on their emulsification properties, through visual assessment^11^. Given the range of possible excipients for SEDDS (i.e., oils, surfactants, cosolvents – all of which may differ in terms of hydrophilicity/lipophilicity, purity, etc.), selection is often narrowed based on generally recognized as safe (GRAS) status. An established tool to facilitate the process of formulation development is the Lipid-based Formulation Classification System (LFCS). The LFCS defines four categories of oral lipid-based formulations according to their compositions, which essentially range from a pure mixture of oils to a combination of exclusively surfactants and cosolvents^6^. While the LFCS relates these compositional ranges to typical properties, it does not eliminate the need to develop bespoke formulations by exploring various excipient combinations. Nonetheless, methods to shift away from the traditional development of SEDDS have emerged, largely employing data-driven tools.

In recent years, there has been significant interest in the integration of artificial intelligence (AI) and machine learning (ML) in pharmaceutical sciences, including drug formulation. These tools have been used in a variety of advanced applications, from the expedited design of polymeric long-acting injectables to engineering peptides for sustained delivery to the eye, and the development of ionizable lipids for lipid nanoparticle delivery of mRNA^12–14^. In the context of oral lipid-based formulations, ML and computational techniques have played a role in early-stage development, notably based on small molecule drug solubility screening^15^. Preliminary ML modeling has been used to predict drug supersaturation in lipid-based formulations and increases in the apparent solubility of drug upon dispersion of SEDDS^16,17^. In these cases, a limited number of formulation compositions (i.e., two representative examples) were explored. Few studies have performed extensive investigations relating to SEDDS compositions. One example includes an approach integrating ML and molecular dynamics to predict self-emulsification regions for SEDDS formulations, which also reported the distribution of excipients in their dataset^18^. However, this study did not identify drugs that were in the formulations in the dataset.

**Fig. 1:**
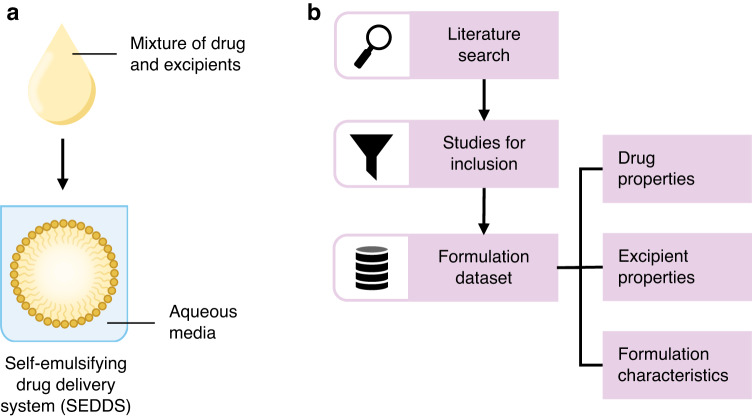
A schematic overview of the study. Graphical illustration of self-emulsifying drug delivery systems (SEDDS), which spontaneously emulsify into colloidal particles upon dispersion of the preconcentrate (i.e., drug-excipient mixture) in aqueous media (**a**). Workflow for the collection of the SEDDS dataset (**b**).

Thus, although SEDDS are a well-established formulation strategy, there are currently no open-access SEDDS datasets with a focus on formulation composition. Here, we present a literature mined SEDDS dataset containing 668 unique formulations, with drug, excipient, and formulation features that may be used to better understand composition patterns or relationships and predict formulation properties (Fig. 1). Our dataset contributes to the development of SEDDS formulations by providing a resource with documented formulations and related information that may serve as a starting point for excipient selection and screening.

## Methods

### Data collection

**Fig. 2:**
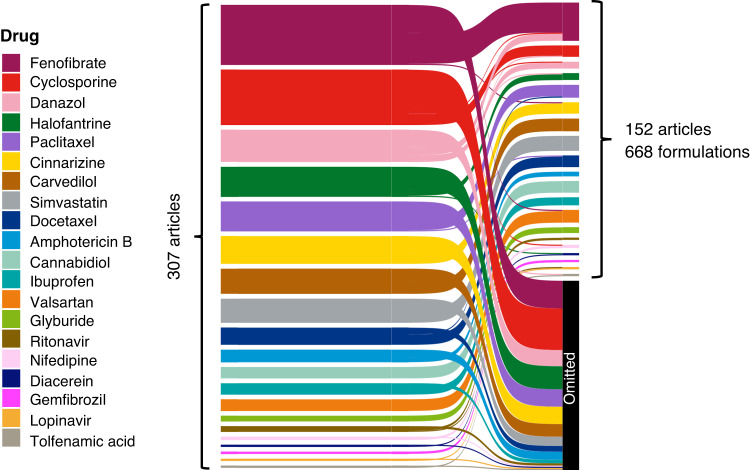
Sankey diagram illustrating the number of articles identified and screened for construction of the SEDDS dataset. An initial pool of 307 articles was selected following a search of the Web of Science database. Manual screening of the articles yielded 152 articles containing 668 unique formulations for inclusion in the dataset. Meandering flows indicate article searches that corresponded to one drug but provided relevant information for a different drug.

All SEDDS formulations in the dataset were collected from published literature. The dataset was constructed based on a search of the Web of Science database covering its inception to March 2023, using the keywords “self-emulsifying drug delivery systems” or “SEDDS” or “SNEDDS” or “SMEDDS” and “drug” from a list of 20 poorly water-soluble drugs (i.e., active pharmaceutical ingredients (APIs)). Search results were limited to articles and filtered by publisher (i.e., Elsevier, Springer Nature, Taylor & Francis, Wiley, MDPI). An initial pool of 307 articles were manually screened, yielding 152 articles that encompassed 668 unique formulations for inclusion in the dataset (Fig. 2). Articles were omitted if they did not provide relevant information, such as insufficient formulation compositional details, description of formulations not corresponding to the drug in question, or a non-unique formulation. The full list of source studies is provided in the source and DOI columns of the sedds_dataset_full.csv file.

Information obtained for an individual sample in the dataset included the identity and relative proportion of the drug, as well as each individual excipient (i.e., oils, surfactants, cosolvents, and other ingredients). Other additives or ingredients were grouped by function (e.g., absorption enhancer, precipitation inhibitor, etc.), as opposed to the individual identity, to facilitate downstream analysis. The proportions of each component for a given formulation were standardized as compositional data, such that they totaled to 100% in units by weight. Additional descriptors included the average particle size (i.e., droplet diameter of SEDDS upon dispersion) and average droplet polydispersity index, where applicable. A manually defined descriptor denoting whether a given formulation was found to be promising in the context of its source article was also included. A formulation was considered to be promising if it was selected for further development and/or exhibited the most favourable properties (i.e., dependent on the original study) from a panel of screened formulations.

The literature-mined dataset was further extended by appending additional features relating to each component of each formulation. Drug physicochemical properties were sourced from DrugBank, while excipient properties were reported according to the literature and supplier or manufacturer information.

### Data preprocessing and feature engineering

To obtain a tractable dataset amenable to downstream analysis and modeling, data cleaning and preprocessing were performed. First, the trade names of excipients were all converted to chemical names, to remove redundancy. For each formulation, the number of oils, surfactants, cosolvents, or other ingredients were counted and converted into a single so-called *SEDDS complexity* feature. This feature was a min-max normalization performed on the total number of ingredients in each formulation (x), according to:

$$x{\prime} =\frac{x-\min (x)}{\max (x)-\min (x)}$$

Furthermore, features describing the oil, surfactant, and cosolvent properties of the formulation were derived from individual component properties. For instance, using the dominant fatty acid within a certain oil (or across mixtures of oils), binary features for whether there is a long aliphatic chain and/or saturated chain described the oil character of a formulation. For surfactant and cosolvent features, weight-average properties were calculated based on the proportions of each excipient in a formulation. The complete procedure and calculations used to generate the dataset are provided in the available R code.

## Data Records

The SEDDS dataset and related data are available in CSV formats on Open Science Framework (OSF)^19^. A summary of the available files is provided in Table 1. Data files contained in the Components folder report all individual drugs and excipients, as well as their associated properties, collated in the final dataset, sedds_df.csv. The data contains 20 drugs, 44 unique oils, 31 unique surfactants, and 17 unique cosolvents. In total, the final cleaned dataset comprised 29 features for 668 SEDDS formulations (Table 2).

**Table 1:** Summary of available data files and their descriptions.

Parent folder	File	Description
Components	sedds_dataset_api.csv	File containing data related to all drugs.
Components	sedds_dataset_oil.csv	File containing data related to all oils.
Components	sedds_dataset_surfactant.csv	File containing data related to all surfactants.
Components	sedds_dataset_cosolvent.csv	File containing data related to all cosolvents.
Components	sedds_dataset_other.csv	File containing data related to all other ingredients.
Initial	sedds_dataset_sankey.csv	File containing data related to the literature search and screening, based on number of articles corresponding to a given drug.
Initial	sedds_dataset_full.csv	File containing data related to all formulations and their characteristics from source studies.
Data	sedds_df.csv	File containing the final, clean dataset.

**Table 2:** List of features in the SEDDS dataset and their related formulation component and description.

Feature	Related component	Type	Description
size	SEDDS	Numeric	*Droplet size*. The average particle size (nm) (i.e., droplet diameter) of the SEDDS upon dispersion.
PDI	SEDDS	Numeric	*PDI*. The average polydispersity index of the SEDDS upon dispersion.
cplx_minmax_norm	SEDDS	Numeric	*SEDDS complexity*. A feature describing the relative complexity of a formulation, by considering the total number of unique excipients, which are min-max normalized.
progressed	SEDDS	Categorical	*Progressed*. Binary variable describing whether the formulation did not progress in a given study (0) or was promising (1) (i.e., progressed past initial screening; investigated for further formulation applications, *in vivo* studies, etc.). A formulation was considered to be promising if it was selected for further development and/or exhibited the most favourable properties (i.e., dependent on the original study) from a panel of screened formulations.
API_prop	Drug	Numeric	*Total API content*. The total content (% w/w) of drug in the formulation.
API_mol_wt	Drug	Numeric	*API molecular weight*. The molecular weight (g/mol) of the drug.
logp_chemaxon	Drug	Numeric	*API logP*. The calculated logP of the drug, sourced from Chemaxon.
API_melt_temp	Drug	Numeric	*API melting point*. The melting point (°C) of the drug.
API_water_sol	Drug	Numeric	*API water solubility*. The estimated water solubility (mg/mL) of the drug, sourced from ALOGPS.
API_polar_sa	Drug	Numeric	*API polar surface area*. The polar surface area (Å^2^) of the drug.
API_rot_bond	Drug	Numeric	*API number of rotatable bonds*. The number of rotatable bonds in the drug molecule.
API_H_bond_donor	Drug	Numeric	*API H-bond donors*. The number of H-bond donors in the drug molecule.
API_H_bond_accept	Drug	Numeric	*API H-bond acceptors*. The number of H-bond acceptors in the drug molecule.
oil_total	Oil	Numeric	*Total oil content*. The total content (% w/w) of oil within the formulation.
o_num	Oil	Numeric	*Number of oils*. The total number of unique oils in the formulation.
o_LC	Oil	Categorical	*Oil long chain*. Binary variable describing whether the character of the oil phase is predominantly medium chain fatty acids (0) or long chain fatty acids (1). Calculated based on the aliphatic chain length of the dominant fatty acid of the dominant oil.
o_sat	Oil	Categorical	*Oil saturated*. Binary variable describing whether the character of the oil phase is predominantly unsaturated (0) or saturated (1). Calculated based on the degree of saturation of the dominant fatty acid of the dominant oil.
surfactant_total	Surfactant	Numeric	*Total surfactant content*. The total content (% w/w) of surfactant within the formulation.
s_num	Surfactant	Numeric	*Number of surfactants*. The total number of unique surfactants in the formulation.
s_HLB	Surfactant	Numeric	*Surfactant HLB*. The weight-averaged hydrophilic-lipophilic balance of surfactants in the formulation.
cosolvent_total	Cosolvent	Numeric	*Total cosolvent content*. The total content (% w/w) of cosolvent within the formulation.
c_num	Cosolvent	Numeric	*Number of cosolvents*. The total number of unique cosolvents in the formulation.
c_mol_wt	Cosolvent	Numeric	*Cosolvent molecular weight*. The weight-averaged molecular weight (g/mol) of the cosolvent.
c_melt_temp	Cosolvent	Numeric	*Cosolvent melting point*. The weight-averaged melting point (°C) of the cosolvent.
c_boil_temp	Cosolvent	Numeric	*Cosolvent boiling point*. The weight-averaged boiling point (°C) of the cosolvent.
c_density	Cosolvent	Numeric	*Cosolvent density*. The weight-averaged density (g/mL) of the cosolvent.
c_viscosity	Cosolvent	Numeric	*Cosolvent viscosity*. The weight-averaged viscosity (mPa·s) at room temperature of the cosolvent.
other_total	Other ingredient	Numeric	*Total other content*. The total content (% w/w) of other ingredients in the formulation.
other_num	Other ingredient	Numeric	*Number of other ingredients*. The total number of unique other ingredients in the formulation.

## Technical Validation

Given the dataset is sourced from the literature, the validity is directly related to the quality of the source studies. Therefore, limitations pertaining to the sparsity and accuracy of reported data, and the influence of publication bias, are to be expected. By including a range of drugs and all their available SEDDS formulations, we strove to impart the dataset with a more representative breadth of samples (i.e., combination of BCS II and IV drugs; some drugs are less amenable to SEDDS formulations than others). Furthermore, studies were assessed for completeness of information and uniqueness of the reported formulation. This ensured all compositional details are available for each sample. All possible features from the source studies were included in the dataset, but there is scope to potentially expand it with additional descriptors, such as structural representations (e.g., for drugs or excipients) for researchers aiming to use the dataset in ML applications. It is notable that droplet size and PDI of SEDDS upon dispersion are not reported in all cases, with only 506 (75.7%) formulations reporting the former and 289 (43.3%) formulations reporting the latter. While this is related to the nature of the data, missing data may be addressed through imputation, the application of synthetic data generation techniques, or by omission.

## Data Availability

All data cleaning and preparation was performed in R (version 4.2.1). The R code used to generate the dataset is available on OSF as a markdown notebook.
